# Detection of seven *Alternaria* toxins in edible and medicinal herbs using ultra-high performance liquid chromatography-tandem mass spectrometry

**DOI:** 10.1016/j.fochx.2021.100186

**Published:** 2021-12-11

**Authors:** Xiangsheng Zhao, Dan Liu, Xinquan Yang, Lei Zhang, Meihua Yang

**Affiliations:** a Key Laboratory of Resources Conservation and Development of Southern Medicine of Hainan Province & Hainan Branch of the Institute of Medicinal Plant Development, Chinese Academy of Medical Sciences and Peking Union Medical College, Haikou 570311, China; b School of Traditional Chinese Medicine, Guangdong Pharmaceutical University, Guangzhou 510006, China; c Institute of Medicinal Plant Development, Chinese Academy of Medical Sciences & Peking Union Medical College, Beijing 100193, China

**Keywords:** *Alternaria*, Herbs, Mycotoxin, Occurrence, UPLC-MS/MS, QuEChERS, AA, acetic acid, ACN, acetonitrile, Alternaria toxins:alternariol, AOH, alternariol mono methylether, AME, altenuene, ALT, altenusin, ALS, altertoxin Ⅰ, ATX-Ⅰ, tenuazonic acid, TeA, tentoxin, TEN, C_18_, octadecyl, CEs, collision energies, EFSA, European Food Safety Authority, ESI, electrospray ionization, FA, formic acid, GCB, graphitized carbon black, LOD, limit of detection, LOQ, limit of quantification, ME, Matrix effect, MeOH, methanol, MCX, Mixed-mode cationic exchange, MRM, multiple reaction monitoring (MRM), PSA, primary secondary amines, relative standard deviation, RSD, QuEChERS, quick, easy, cheap, effective, rugged, safe, SPE, solid phase extraction, TCMs, traditional Chinese medicines, UPLC-MS/MS, ultra-high performance liquid chromatography-triple quadrupole mass spectrometry

## Abstract

*Alternaria* mycotoxins are ubiquitous mycotoxins that contaminate food and animal feed. Here, an UPLC-MS/MS was developed and used for the detection of seven *Alternaria* mycotoxins in 19 different edible and medicinal herbs. Extensive optimization resulted in a simple and convenient sample preparation procedure with satisfactory extraction and a lower matrix effect. LOQs ranged from 0.01 to 2.0 ng/mL. Recoveries varied between 71.44% and 112.65%, with RSD less than 12%. The method was successfully applied for use in the mycotoxin analysis of 260 samples. A high percentage (28.46%) of samples were contaminated by 1–5 mycotoxins. Alternariol mono methylether was the predominant mycotoxin with high percentage of positive samples (37.5%), followed by alternariol (22.5%), alternariol (17.5%), tentoxin (10.83%), altertoxin Ⅰ (7.5%), and altenusin (4.17%). Collectively, the natural incidence data obtained from this study will help with better, validated risk assessments and efforts towards more comprehensive, future regulation.

## Highlights

• A modified QuEChERS-UPLC-MS/MS method was established to investigate alternaria mycotoxins.
• The method was applied to 260 edible and medicinal herb samples.
• 28.46% of samples were contaminated by at least one toxin.
• AME with a high occurrence in analyzed herbs.

## Introduction

Globally, the contamination of food with environmental pollutants, drug and chemical residues, microbial pathogens, and a wide array of other anthropogenic and natural types of contaminants poses a serious public health concern. In this context, mycotoxins—a class of food contaminants of natural origin—have received increasing interest, owing to their potential toxic effects on human and animal healthy as well as their high prevalence in food (Crudo et al., 2021). To date, the majority of mycotoxins relevant to agriculture are produced by fungi belonging to one of four genera—*Aspergillus, Fusarium, Penicillium*, and *Alternaria* (Goessens et al., 2021). Of these, *Alternaria* is a cosmopolitan fungal genus that includes saprophytic, endophytic, and pathogenic species that are ubiquitous across many natural and anthropogenic environments (Sivagnanam et al., 2017). As pathogens, they affect many agricultural products including grains, oil seeds, spices, fruits, vegetables, and herbs. They may then enter the food chain through these routes (Mujahid et al., 2020).

Due to their growth at low temperature, *Alternaria* fungal species are responsible for spoilage of commodities during refrigerated transport and storage. Additionally, *Alternaria* microfungi are capable of growing at low temperatures and are responsible for food spoilage during refrigerated transport and/or storage (Fan, Cao, Liu, & Wang, 2016). *Alternaria* toxins (ATs) are secondary metabolites produced by *Alternaria* species. The most common of these species is *Alternaria alternate* but also includes *Alternaria tenuissima* and *Alternaria infectoria*. (Fan et al., 2016). Although more than 70 ATs are produced, only approximately 30 mycotoxins have been isolated whose respective chemical structures place them in different classes (Xing et al., 2020).

*Alternaria* mycotoxins are broadly classified into dibenzopyrone derivatives, perylene derivatives, and tetramic acid derivatives. Of these, alternariol (AOH), alternariol monomethyl ether (AME), tenuazonic acid (TeA), tentoxin (TEN), and altenuene (ALT) are considered the most important ATs (Qiao et al., 2020, Zhou et al., 2017). Past work has focused on using *in vitro* and *in vivo* assessments to better understand the toxicity, mutagenicity, and genotoxicity of these ATs (Lehmann et al., 2006, Ostry, 2008, Schrader et al., 2001, Somma et al., 2019). Moreover, work in Linxian, China indicated that ATs may be one of the etiological factors responsible for its rate of human esophageal cancer (Liu et al., 1992). In view of the potential toxicity of ATs, the European Food Safety Authority (EFSA) issued the threshold of toxicological concern (TTC) for AME, AOH, TeA, and TEN (Arcella et al., 2016, EFSA Panel on Contaminants in the Food Chain, 2011). Despite this, there are currently no regulatory limits or monitoring guidelines established for ATs in either food or plant products.

Some edible and medicinal herbs have been used to prevent and cure human disease for several millennia. Moreover, these herbs are also used as spices, additives, and/or edible foods. Unfortunately, they may be contaminated by ATs before harvesting, which results in safety concerns. At present, the occurrence of ATs has been reported mainly in fruits and fruit products (De Berardis et al., 2018, Juan et al., 2017), vegetables (Dong et al., 2019; Theresa Zwickel et al., 2016), and cereals (Zhou et al., 2017; Hannes Puntscher et al., 2019). Relatively limited data are available on the occurrence of ATs in herbs, as previous work has not routinely assessed levels of these toxins in edible and medicinal herbs (Xing et al., 2020, Wu et al., 2021). Recently, *Alternaria* toxins have been referred to as “emerging” mycotoxins found in medicinal plants. Therefore, finding a reliable, analytical methodology for monitoring ATs that is also applicable to herbs is greatly needed.

There is a growing tendency when conducting a multi-mycotoxin analysis to develop rapid liquid chromatography tandem mass spectrometry (LC-MS/MS) methods with minimum sample treatments. However, the complex matrix and different physical and chemical properties of ATs have caused significant challenges to analytical researchers in obtaining widespread occurrence data and various contamination levels of ATs in medicinal plants.

Quick, easy, cheap, effective, rugged, and safe (QuEChERS)-based methodologies have been used as an effective and convenient sample preparation procedure. They have also been extensively used in risk monitoring of several kinds of mycotoxins in traditional Chinese medicines (TCMs) (Zhang et al., 2018). Given this, the aim of this paper was to develop a modified QuEChERS approach coupled with a UPLC-MS/MS method for the simultaneous determination of seven ATs—alternariol, alternariol mono methylether, altenuene, altenusin, tenuazonic acid, altertoxin Ⅰ, and tentoxin (Fig. S1). Moreover, this approach was used to evaluate the presence of these contaminants in edible and medicinal herbs. Representative matrices (19 in total) were selected in view of their higher consumption in China and included nutmeg, Alpinia officinarum Rhizoma, Citri reticulatae Pericarpium, and Crataegi Fructus.

## Materials and methods

### Chemical, reagents, and materials

Acetonitrile (ACN), methanol (MeOH) formic acid (FA), acetic acid (AA), ammonium formate, and ammonium acetate—all of HPLC-grade—were purchased from Merck (Darmstadt, Germany). Analytical grade ammonium carbonate ((NH_4_)_2_CO_3_, 99.99%) was obtained from Macklin (Shanghai, China). Ultrapure water was prepared using a Milli-Q system (Millipore Corporation, Bedford, MA, USA). For the cleanup of extracts, were used primary secondary amine (PSA), graphitized carbon black (GCB), octadecyl (C_18_), and aminopropyl (–NH_2_) sorbents, all purchased from Agela Technologies (Tianjin, China), Magnesium sulfate (MgSO_4_) and sodium chloride (NaCl) were obtained from Xilong Chemical (Guangdong, China). Mixed-mode cationic exchange (MCX) was ordered from Biocomma (Shenzhen, China). All other chemicals were of analytical grade. An Oasis HLB SPE cartridge (60 mg, 3 cc) was purchased from Waters Corp. (Milford, MA, USA).

Analytical standards of *Alternaria* mycotoxins alternariol (AOH), alternariol mono methylether (AME), altenuene (ALT), altenusin (ALS), altertoxin Ⅰ (ATX-Ⅰ), tenuazonic acid (TeA), and tentoxin (TEN) were all obtained from Pribolab (Singapore) and were more than 98% pure. The chemical structures of ATs are shown in Fig. S1. The isotopically labelled internal standards (IS) [^13^C_14_]-AOH and [^13^C_10_]-TEA were purchased from Pribolab (Singapore).

Nineteen species of edible and medicinal herbs (Nutmeg, Alpinia officinarum Rhizoma, Citri reticulatae Pericarpium, Crataegi Fructus, Jujubae Fructus, Lycii Fructus, Chaenomelis Fructus, Menthae Haplocalycis Herba, Mori Fructus, Schisandrae Chinensis Fructus, Corni Fructus, Coicis Semen, Cassiae Semen, Gardeniae Fructus, Nelumbinis Semen, Trichosanthis Pericarpium, Lilii Bulbus, Longan Arillus, Zingiberis Rhizoma Recens) were all purchased from herbal markets or pharmacies in Anguo (Hebei Province), Bozhou (Anhui Province), or Yulin (Guangxi Province), etc. All samples were stored in a dry and well-ventilated place away from direct sunlight. Prior to the analysis, all samples were ground to homogenous size using a mill and sized through a 50-mesh sieve.

### Instrumentation

A Waters ACQUITY UPLC^TM^ (Milford, MA, USA) system was interfaced to a triple quadrupole MS (TQD, Waters, Manchester, UK) using an orthogonal Z-spray electrospray ionization (ESI) interface. The chromatographic separation was conducted on a BEH C_18_ column (1.7 µm, 2.1 × 100 mm, Waters Corp.), and the column temperature was set to 40 °C. The mobile phase consisted of methanol (A) and 0.15 mmol/L of (NH_4_)_2_CO_3_. The separation was performed at a flow rate of 0.25 mL/min, with a gradient elution program as follows: 0 min:5％ A; 6 min: 95% A; 8 min: 5% A; 10 min; 5% A. The injection volume was 3.0 µL. The post-time for column re-equilibration was set to 3 min between consecutive injections. Based on the structural properties of target ATs, both the ESI^±^ and ESI^-^ modes were applied. The parameters were as follows: Capillary voltage, +3.5 kV/−2.5 kV; source temperature, 150 °C; desolvation temperature, 400 °C; desolvation gas flow, 800 L/h; and cone gas flow, 50 L/h. Nitrogen was used as the cone and desolvation gas, and argon was used as the collision gas. Optimal collision energies (CEs) and cone voltages selected for each transition and retention time are shown in Table S1. The MassLynx^TM^ 4.1 software was used for data acquisition and processing.

### Preparation of standard solutions

Each standard was weighed and dissolved in methanol to prepare stock solutions of 100 μg/mL and stored in darkness at −20 °C. The mixed standard solution was prepared by stepwise dilution of individual standard stock solution with methanol. A series of mixed standard working solutions for calibration standards were prepared by diluting the mixed standard solution with a same amount of IS.

### Sample preparation

The sample powder (1.0 g) was accurately weighed accurately into a 50 mL centrifuge tube and 5.0 mL of H_2_O was added. The tube was vortexed for 1.0 min and placed aside for 20 min. Afterward, 5.0 mL of 1.0% FA in ACN was added and immediately vortexed for 10 min. Then, 2.0 g of magnesium sulfate and 0.5 g of sodium chloride were added and immediately vortexed for 1.0 min. This was done to enhance the partition of the mycotoxins into the organic layer. The tube was subsequently centrifuged at 10,000 rpm for 5.0 min. Then, 2.0 mL of the supernatant was transferred to a clean tube and evaporated under a soft stream of nitrogen gas at 40 °C. The residue was re-dissolved with 1.0 mL ACN/H_2_O (1:1, *v/v*), and centrifuged at 13,000 rpm for 15 min. Finally, 3.0 µL of the resulting solution was injected into the UPLC-MS/MS for analysis.

### Method validation

The developed method was validated according to a validation guideline produced by the International Conference on Harmonization (ICH) (2005). Investigated parameters included linearity, precision, recovery, limit of quantification (LOQ), and accuracy. The linearity was evaluated by least squares regression analysis using calibration curves, which were plotted by peak area ratios of the analyte to the IS against the corresponding analyte concentrations. The LOD, LOQ, and precision (intra- and inter-) were performed on three different types of non-contaminated herbs: Nutmge, Coix seed, and Alpinia officinarum Rhizoma. The LOQ was determined as the analyte concentration at which the signal-to-noise ratio (S/N ratio) was greater than 10. Precision was assessed using the relative standard deviation (RSD, %) of target ATs after extraction of the three blank representative matrices. For the recovery assay, three, non-contaminated herbs were selected and spiked with low, intermediate, and high levels of mixed standards (low levels: 1.0 μg/kg for AME, 2.5 μg/kg for AOH, TEN and ATX-1, 5 μg/kg for ALS, TEA, and ALT; middle levels: 5.0 μg/kg for AME, 12.5 μg/kg for AOH, TEN, and ATX-1, 25 μg/kg for ALS, TEA and ALT; high levels: high level: 25 μg/kg for AME, 62.5 μg/kg for AOH, TEN and ATX-1, 125 μg/kg for ALS, TEA, and ALT). Samples were extracted with the proposed procedure and quantified using calibration curves constructed in solvent. The matrix effect (ME) was calculated as follow: ME (%)=(A_1_/A_2_) × 100, where A_1_is the peak area ratio of the ATs and the IS for a blank sample with standard addition after extraction and A_2_ is the peak area ratio of the ATs and IS for a corresponding standard solution.

## Results and discussion

### Optimization of UPLC-MS/MS conditions

The optimization of both the electrospray ionization mode and tandem mass spectrometric parameters were achieved by infusion of reference standard solutions (individual toxin in methanol, 1 μg/mL) using a syringe pump. To mimic actual infusion conditions, a flow rate of 5–10 μL/min tune solution was combined with a UPLC flow of 0.25 mL/min. For each target toxin, the optimized parameters included cone voltage, collision voltage, qualitative ion pairs, and quantitative ion pairs. Experiments were conducted in both positive and negative ESI modes. Most of the analytes (ALS, AOH, ATX-Ⅰ, TEN, and AME) were detected as parent ions with high sensitivity in ESI negative ion mode. Only TEA and ALT were not detected in this way, which showed parent ions with higher intensity in positive mode.

Full-scan acquisitions were performed to obtain parent ion and optimum cone voltage. The toxins of ALS, AOH, ATX-Ⅰ, TEN, and AME formed abundant [M−H]^−^, while the [M + H]^±^ adduct was predominant for TEA and ALT. Once the parent ion was selected, daughter ion scan acquisitions were conducted at different collision energies (CE) to select their suitable daughter ions and optimum CE. Two transitions between the parent ion and the most abundant daughter ions were monitored for the identification of each target toxin; the ion pair with relatively higher intensity was selected for quantification. The optimized MS parameters for these target toxins are shown in Table S1.

**Fig. 1:**
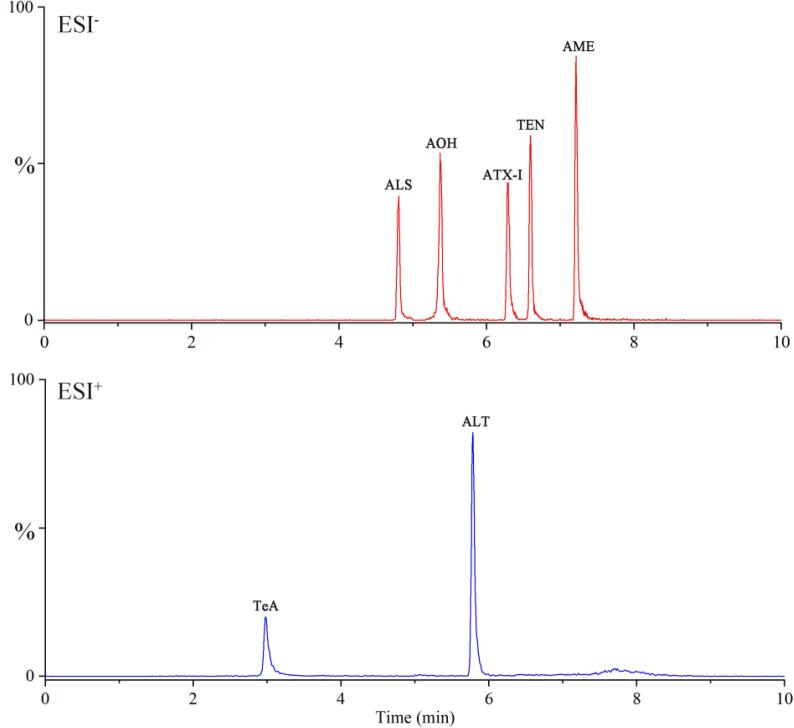
UPLC-MS/MS MRM chromatograms of 7 *Alternaria* toxins.

Then, in order to obtain good separation and response, several factors that influenced retention and chromatographic efficiency were optimized, including column, mobile phase, flow rate, and column temperature. Based on peak shape, resolution, and retention time, C_18_ column was retained as the most suitable column in reversed phase UPLC-MS/MS system for multi-mycotoxin analysis (Wang et al., 2016). Given this, we next investigated the separation performances of 7 target mycotoxins on a Waters BEH C_18_ (2.1 mm × 100 mm, 1.7 µm) with different mobile phases. Better resolution and sensitivity were obtained when using MeOH as the eluent compared with ACN. In addition, different modifiers (e.g., formic acid, acetic acid, ammonium formate, ammonium acetate, and ammonium carbonate) in the mobile phase were tested. Results indicated that the acid system (FA, AA), ammonium formate, and ammonium acetate system had severe ionization suppression effects on target toxins (e.g., AOH, AME, and TEA) detected in positive ionization mode and negative ionization mode, respectively. Additionally, TEA had serious tailing peaks under either the formic acid or acetic acid systems. Peak shapes and the overall responses for the 7 target mycotoxins in ammonium carbonate were notably improved. This result was consistent with previous studies that also used ammonium carbonate as an additive in the mobile phase for the analysis of ATs (De Berardis et al., 2018, Qiao et al., 2020). Given these findings, the final selection was a gradient elution using a mobile phase containing both MeOH and 0.15 mM ammonium carbonate in water. In addition, the effects of flow rate (0.1–0.5 mL/min) and column temperature (30–50 °C) were evaluated. The best chromatographic resolution was achieved when the column temperature was set at 40 °C and the elution flow was performed at 0.25 mL/min. Under these optimized instrument conditions, MRM chromatograms of 7 mycotoxins in standard solutions were obtained and are shown in Fig. 1.

### Optimization of sample preparation

#### Selection of extraction solvent

Sample preparation before assessing the 7 ATs in edible and medicinal herbs is a critical step. For optimized experiments, nutmeg was used as a blank matrix, and a test sample was prepared by spiking a standard mixture (5.0 μg/kg for AME, 12.5 μg/kg for AOH, TEN and ATX-Ⅰ, 25 μg/kg for ALS, TEA and ALT) into the blank. To achieve the most efficient extraction for the target mycotoxins and to minimize the interference of the co-extraction compounds, the effect of different extraction solvents was also investigated. The recoveries of the target toxins increased after adding water to the sample in the pre-experiment step. This observation was consistent with previous reports, as it enabled the release of analytes that were bound to the matrix (Dzuman et al., 2014). Thus, 5.0 mL of H_2_O was added to all samples before organic solvent extraction.

**Fig. 2:**
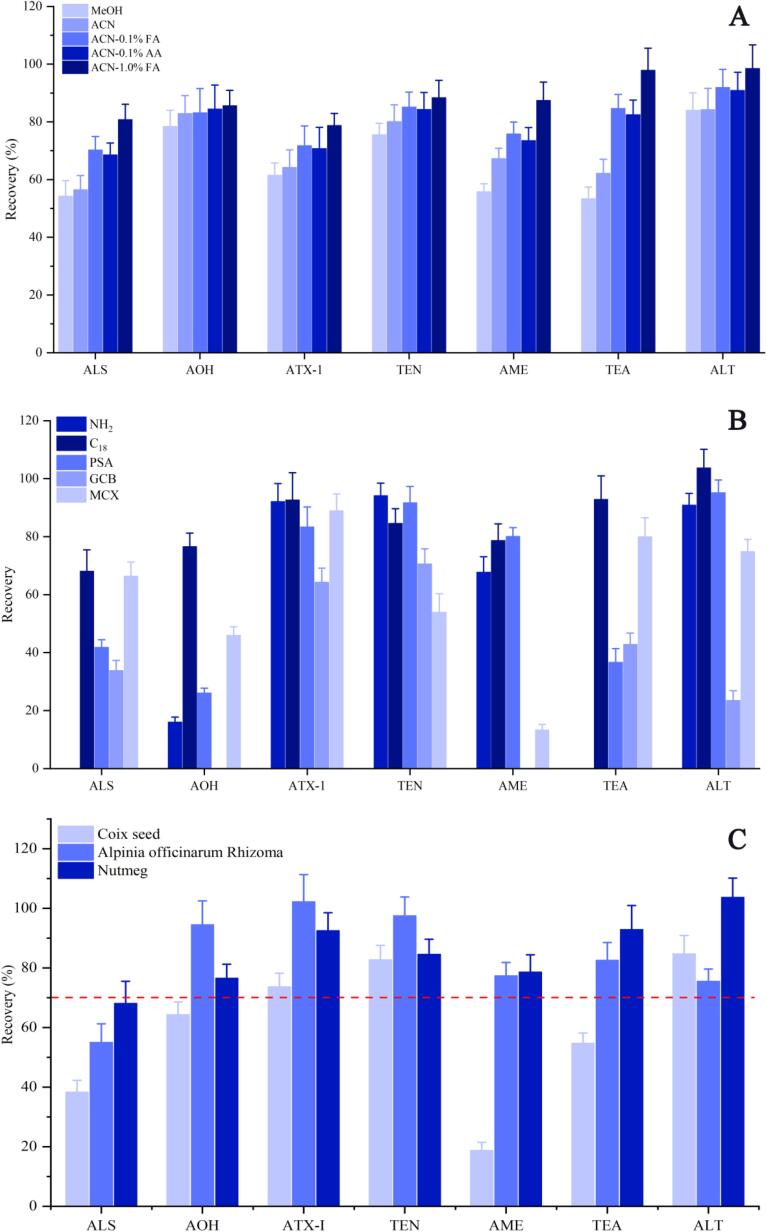
Effects of extraction solvent (A), different sorbents (B) and matrices (C) on the recoveries of target toxins.

The common organic solvents for extracting ATs were MeOH, ACN, and ethyl acetate. Ethyl acetate extracted a large amount of pigment, owing to its excellent lipid solubility (Dong et al., 2019). The extraction efficiencies of ACN and MeOH with different concentrations of acid were also evaluated. As shown in Fig. 2A, the extraction efficiencies of ALS, AME, and TEA with MeOH were all lower than the recoveries obtained with ACN. The addition of acid (FA or AA) to the extraction solvent increased the recoveries of all target mycotoxins; this was especially true for the acidic toxins (TEA). Acid type was not significant for the extraction efficiencies of mycotoxins at low concentrations. Sensitivity remained low with increasing AA concentration (1.0%). When 1.0% FA was used instead of AA, the highest extraction efficiency was achieved, with recoveries ranging from 78.73% and 98.45%. However, extraction efficiency was decreased—especially for TEA—while FA concentration was above 1.0%. Collectively, these results were similar to those of previous work (Zhou et al., 2019). Finally, ACN with 1.0% FA was chosen as the extraction solvent for the following step.

#### Comparison of SPE purification and QuEChERS-dSPE clean-up

Matrix effects resulting from co-extracted matrix components may affect—in either a suppressive or enhancing capacity—the ionization efficiency of the analytes. This could lead to lower sensitivity for some mycotoxins and subsequent erroneous quantitative results (Wang et al., 2016). To achieve the best extraction efficiency, the clean-up methods of SPE and QuEChERS-dSPE were next evaluated. Detailed procedures regarding each preparation method are described in Supplementary Material S1. A previous study reported satisfactory recoveries (greater than 80%) of five ATs (ALT, AME, AOH, TEA, TEN) after clean-up using HLB SPE cartridge for a multi-mycotoxin (24 mycotoxins) analysis in Coix seed (Wu et al., 2021). In our study, HLB SPE was first tried; however, the results showed that recoveries of ALS, ATX-Ⅰ, and TEA were only between 45.36% and 63.03% (Fig. S2). Therefore, we determined that HLB SPE clean-up was not a suitable method for analysis of the 7 ATs in this study.

Next, QuEChERS-dSPE clean-up was evaluated. As sorbent is the main experimental factor, it was investigated in the first step. Five commonly used adsorbents including octadecylsilyl (C_18_), primary secondary amine (PSA), aminopropyl (–NH_2_), mixed-mode cationic exchange (MCX), and GCB were compared using recovery as an index. QuEChERS-dSPE clean-up results (Fig. 2B) indicated that four sorbents (PSA, –NH_2_, MCX, and GCB) were not suitable for the simulations clean-up of all target mycotoxins. As formerly reported (Anastassiades et al., 2003), PSA and –NH_2_ mainly interacted with chemicals by hydrogen bonding; in this way, they removed similar types of components, including fatty acids, other organic acids, and—to some extent—various sugars and pigments. Unfortunately, using –NH_2_ resulted in an almost complete absorption of ALS and TEA. More specifically, recoveries for both were 0 and the recovery of AOH was 16.01%. Moreover, unacceptable recoveries were also obtained for ALS, AOH, and TEA (26.01%–41.77%) while using PSA. GCB effectively removed the pigment in the matrix. However, the recovery values of ALS (33.76%), AOH (0), AME (0), TEA (42.78%), and ALT (23.44%) were all lower than 50%. The poor extraction efficiencies in the present work might be related to the *π-π* interaction through the *sp^2^* hybrid orbitals of GCB and the planar aromatic compounds (e.g., AOH, AME, ALT.) (Guo et al., 2019). Mycotoxin adsorption on GCB has also been reported in other work (Dong et al., 2019). In addition, MCX showed poor recoveries for AOH (45.93%), AME (13.28%), and TEN (53.88%).

In contrast, C_18_ are most commonly used for mycotoxin purification (Zhao et al., 2021, Wei et al., 2019), and the extraction efficiency using this approach was significantly higher than other sorbents. Except for ALS (68.04%), recoveries were all in the range of 76%–104%. The above results indicated that C_18_ could be selected as an adsorbent in QuEChERS method for nutmeg. After this determination, the amount of C_18_ used for target toxins was further optimized. As shown in Fig. S3, 100 mg C_18_ provided better recovery values than other tested amounts (50 mg, 150 mg, and 200 mg). Given this, 100 mg C_18_ adsorbent was selected as a suitable adsorbent for cleaning-up the extract of nutmeg prior to UPLC-MS/MS analysis of ATs.

To determine if the feasibility of the proposed methods could cover a variety of edible and medicinal herbs, the recoveries of ATs spiked in three representative matrices (nutmeg, Coix seed, and Alpinia officinarum Rhizoma) were compared. The seven ATs standards were added into the matrices at concentrations of 5.0 μg/kg for AME, 12.5 μg/kg for AOH and ATX-1, and 25 μg/kg for ALS, TEA and ALT. The sample preparation procedure was the same as described in Section 2.4, and 100  mg C_18_ used as sorbent. As shown in Fig. 2C, the optimized QuEChERS method was not suitable for the extraction of Coix seed, with lower recoveries of ALS (38.29%), AME (18.76%), and TEA (54.69%). For Alpinia officinarum rhizoma and nutmeg, recovery values were generally above 70%. Exceptions included ALS in Alpinia officinarum rhizoma (54.98%) and nutmeg (68.04%). Therefore, using C_18_ as the clean-up sorbent in the QuEChERS-dSPE clean-up method was not useful across a broad range of edible and medicinal herbs.

#### Comparison of One-step and modified QuEChERS

**Fig. 3:**
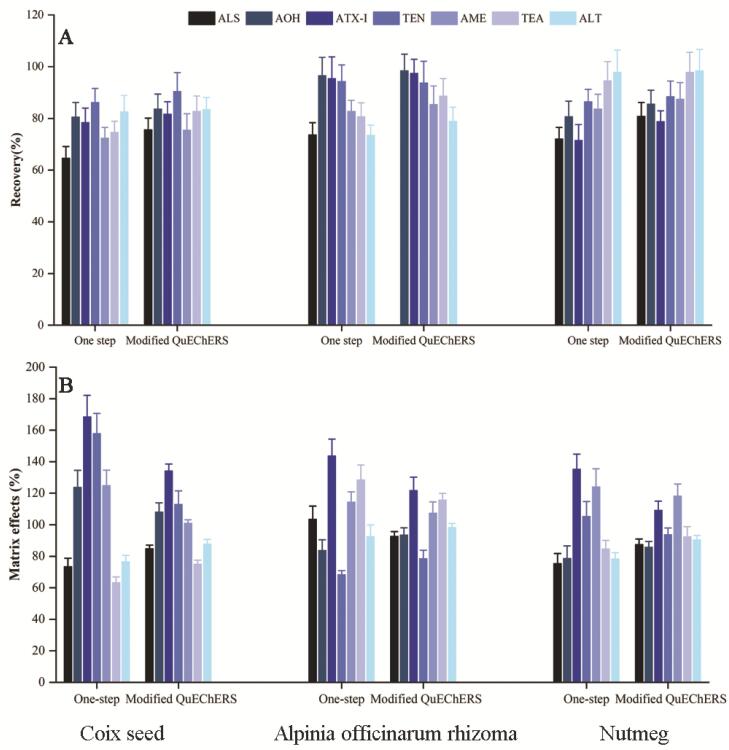
Recoveries (A) and matrix effects (B) of the 7 toxins in Coix seed, Alpinia officinarum rhizome and Nutmeg by two sample preparations.

Finally, one-step extraction without purification (Supplementary Material S1) and a modified QuEChERS (as described in section 2.4) were performed for nutmeg, Coix seed, and Alpinia officinarum rhizoma based on recovery and matrix effects. As shown in Fig. 3, all analytes were cleaned up by a modified QuEChERS method and achieved satisfactory recovery values across a range of 78.73–98.45% in nutmeg, 75.45–90.43% in Coix seed, and 78.52–98.46% in Alpinia officinarum rhizoma. The influence of the matrix on the mass spectrometric detection differed depending on the matrix/sample type. Co-eluting components of the matrix either reduced or enhanced the analyses’ signals. Generally, ME from 80% to 120% indicated the signal had been suppressed (80%–100%) or enhanced (100%–120%) and was still acceptable; comparatively, ME values less than 80% or greater than 120% indicated strong matrix effects (Xing et al., 2020). The MEs of different blanks of nutmeg, Coix seed, and Alpinia officinarum rhizoma were compared using two sample preparation methods as shown in Fig. 3. As indicated, the signal enhancement/suppression was prominent for target toxins using a one step extraction without clean-up, with ME ranging from 63.23% to 168.47%. Conversely, the ME effects were notably reduced when following a modified QuEChERS method, with ME ranging from 74.96% to 134.14%. These results indicated that a salting-out step with anhydrous MgSO_4_ and NaCl contributed to higher recoveries and lower matrix effects. Therefore, the modified QuEChERS method was used to determine ATs in different edible and medicinal herbs.

### Method validation

To check the suitability of this method for determining target toxins in edible and medicinal herbs, linear range, LOD, LOQ, precision, and recovery were all evaluated as described in Section 2.3. As shown in Table 1, the calibration curves presented good linearity for all mycotoxins over the studied ranges with coefficients of determination *R^2^* higher than 0.9980. The values of LOQ ranged from 0.01 to 1.0 ng/mL for nutmeg, 0.02–1.5 ng/mL for Coix seed, and 0.02–2.0 ng/mL for Alpinia officinarum rhizoma. These results showed that the method was sufficiently sensitive to determine the 7 mycotoxins in the all the matrices. Intra- and inter-day precision for ATs at middle concentration levels in different matrices were less than 10.0%, except for TEA in nutmeg. The mean recoveries were 75.29%–112.65%, 72.73–102.52%, and 71.44%–108.33% for nutmeg, Coix seed, and Alpinia officinarum rhizoma, respectively, and the RSDs (%) were all less than 12% (Table 2).

**Table 1:** Linearity, LOD, LOQ and precisions of *Alternaria* toxins in different matrices.

Toxins	Calibration curves	*R^2^*	Linear range (ng/mL)	Nutmeg				Coix seed				Alpinia officinarum Rhizoma			
				LOD (ng/mL)	LOQ (ng/mL)	Precision (RSD, %)		LOD (ng/mL)	LOQ (ng/mL)	Precision (RSD, %)		LOD (ng/mL)	LOQ (ng/mL)	Precision (RSD, %)	
						Intra-day	Inter-day			Intra-day	Inter-day			Intra-day	Inter-day
ALS	*Y* = 0.033*X* + 0.0559	0.9987	2.0–200	0.35	1.0	3.87	6.43	0.45	1.5	5.65	8.67	0.40	1.50	4.15	7.74
AOH	*Y* = 0.1937X + 0.3162	0.9985	1.0–100	0.01	0.04	4.75	8.57	0.08	0.30	4.59	9.58	0.01	0.05	3.28	5.29
ATX-1	*Y* = 0.1238*X* + 0.1347	0.9983	1.0–100	0.09	0.30	3.66	3.89	0.07	0.25	4.64	6.72	0.08	0.25	3.06	4.76
AME	*Y* = 1.6538*X* + 0.5305	0.9996	0.2–40	0.003	0.01	2.24	4.87	0.004	0.02	5.71	7.98	0.005	0.02	2.58	6.42
TEN	*Y* = 0.1484*X* + 0.029	0.9998	1.0–100	0.09	0.30	5.68	9.36	0.06	0.2	4.44	7.65	0.01	0.05	4.81	8.32
TEA	*Y* = 0.0176*X* − 0.0755	0.9992	2.0–200	0.30	1.0	6.24	10.76	0.35	1.2	5.23	8.46	0.40	1.5	4.23	3.87
ALT	*Y* = 0.3986*X* − 0.0754	0.9998	2.0–200	0.25	0.80	4.58	8.05	0.30	1.0	3.68	6.97	0.45	2.0	5.04	5.87

**Table 2:** Recoveries of *Alternaria* toxins in different blank matrices (*n* = 3).

Toxins	Spiked levels	Nutmeg		Coix seed		Alpinia officinarum Rhizoma	
		Recovery (%)	RSD (%)	Recovery (%)	RSD (%)	Recovery (%)	RSD (%)
ALS	Low	76.37	4.32	72.73	3.68	71.44	4.76
	Middle	80.78	7.34	75.56	4.52	78.52	6.35
	High	79.56	8.23	78.29	4.18	81.38	8.21
AOH	Low	75.29	8.58	86.17	10.29	104.35	7.89
	Middle	85.54	5.37	83.66	5.76	98.46	6.35
	High	82.91	7.39	90.24	6.18	93.56	5.85
ATX-Ⅰ	Low	83.82	3.43	78.21	2.44	108.33	9.61
	Middle	78.73	4.17	81.65	4.78	97.44	5.38
	High	89.35	9.43	84.65	8.03	91.04	6.28
TEN	Low	84.29	4.22	95.87	5.17	96.36	3.79
	Middle	88.37	6.03	90.43	7.29	93.65	8.44
	High	96.21	3.32	102.52	9.25	89.51	5.74
AME	Low	93.88	11.34	77.39	3.73	83.24	6.38
	Middle	87.43	6.36	75.45	6.37	85.43	7.08
	High	82.67	7.29	84.26	5.18	88.45	9.15
TEA	Low	85.81	4.37	83.21	7.27	79.47	4.89
	Middle	97.84	2.69	82.66	6.01	88.64	6.73
	High	90.76	7.81	88.47	5.42	82.54	5.37
ALT	Low	112.65	6.76	80.06	3.25	72.88	10.46
	Middle	98.45	8.22	83.45	4.62	78.86	5.45
	High	104.33	4.73	88.63	7.35	80.23	7.93

Low levels: 1.0 μg/kg for AME, 2.5 μg/kg for AOH, TEN and ATX-1, 5 μg/kg for ALS, TEA and ALT; Middle levels: 5.0 μg/kg for AME, 12.5 μg/kg for AOH, TEN and ATX-1, 25 μg/kg for ALS, TEA and ALT; High levels: high level: 25 μg/kg for AME, 62.5 μg/kg for AOH, TEN and ATX-1, 125 μg/kg for ALS, TEA and ALT;

### Real sample analysis

The developed method was next used to determine the natural occurrence and level of ATs in 19 edible and medicinal herbs that are widely used in China. Figs. S4–S8 show the MRM chromatograms that positively identified mycotoxins in different herbs. Table 3 shows the contaminate levels of each AT and their positive number in the different herbs. Six herbs (Gardeniae Fructus, Nelumbinis Semen, Trichosanthis Pericarpium, Lilii Bulbus, Longan Arillus, and Zingiberis Rhizoma Recens) were negative for all tested mycotoxins. Moreover, no sample simultaneously contained all 7 target ATs. Collectively, 74 of 260 samples (28.46%) were contaminated by at least one mycotoxin; however, ALT was not detected in any sample. As shown in Fig. S9, high co-occurrence of mycotoxins was verified in these samples, and the number of ATs in positive samples was different. Therefore, multi-toxin pollution of edible and medicinal herbs was a common phenomenon.

**Table 3:** Occurrence and levels of *Alternaria* toxins in edible and medicinal herbs.

**Edible and medicinal herbs**	**S.N.**	**P.N.**	**Range (μg/kg) (P.N.)**						
			**ALS**	**AOH**	**ATX-Ⅰ**	**TEN**	**AME**	**TEA**	**ALT**
Nutmeg	30	6	169.19(1)	/	14.00–46.10 (5)	/	2.8 (1)	/	/
Alpinia officinarum Rhizoma	25	14	/	44.24–177.91 (10)	/	/	0.94–15.97 (11)	28.62–47.29 (2)	/
Citri reticulatae Pericarpium	11	3	/	79.14–82.25 (2)	/	5.41 (1)	/	40.76–963.95 (3)*	/
Crataegi Fructus	9	3	42.4–58.76 (2)	8.23 (1)	/	7.44–22.52 (2)	0.32–4.64 (3)	40.76 (1)	/
Jujubae Fructus	15	2	/	/	/	/	/	44.03–100.5 (2)	/
Lycii Fructus	13	1	/	/	/	5.99 (1)	/	/	/
Chaenomelis Fructus	8	3	34.22 (1)	Tr–9.34 (2)	/	18.17 (1)	Tr–4.73 (3)	/	/
Menthae Haplocalycis Herba	15	9	/	Tr–193.04 (6)	48.83–63.17 (2)	/	0.49–4.50 (9)	/	/
Mori Fructus	10	8	Tr (1)	/	14.34 (1)	4.34 (1)	0.41–3.35 (8)	276.46–2102.31 (3)*	/
Schisandrae Chinensis Fructus	12	5	/	Tr (1)	/	5.99–14.11 (3)	Tr–0.85 (2)	512.62 (1) *	/
Corni Fructus	10	8	/	8.23–24.41 (4)	/	/	0.05–4.34 (8)	/	/
Coix seed	20	5	/	24.84 (1)	21.85 (1)	Tr (1)	/	136.91–198.05 (2)	/
Cassiae Semen	18	7	/	/	/	Tr-5.99 (3)	/	47.76–201.31 (7)	/
Gardeniae Fructus	13	0	/	/	/	/	/	/	/
Nelumbinis Semen	14	0	/	/	/	/	/	/	/
Trichosanthis Pericarpium	10	0	/	/	/	/	/	/	/
Lilii Bulbus	12	0	/	/	/	/	/	/	/
Longan Arillus	10	0	/	/	/	/	/	/	/
Zingiberis Rhizoma Recens	5	0	/	/	/	/	/	/	/
Total number	260	74	5	27	9	13	45	21	/
Concentration range (μg/kg)			Tr–169.19	Tr–193.04	14.00–63.17	Tr–22.52	Tr–15.97	28.62–2102.31	/

Tr: <LOQ; / = not detected; S.N., Sample number; P.N., Positive number; *, The content exceed the linear range.

Within the herbs in the present study the incidence of positive samples was higher for AME (positive number, 45), with levels ranging from trace (<LOQ) to 15.97 μg/kg (Alpinia officinarum rhizoma). TEA was present in 8 herb samples, from which Mori Fructus herb showed the highest level (2102.31 μg/kg). AOH and TEN were also detected in 8 herb samples, with levels ranging from trace to 193.04 μg/kg (Menthae Haplocalycis Herba) and from trace to 22.52 μg/kg (Crataegi Fructus), respectively. Regarding ATX-1 and ALS, the two mycotoxins was both found in four herbs samples, with positive levels ranging from 14 μg/kg (nutmeg) to 63.17 μg/kg (Menthae Haplocalycis Herba) and from trace to 169.19 μg/kg (nutmeg), respectively. Despite the high detection rate of AME, their residual levels were lower than TEA (28.62–2102.31 μg/kg). This was similar to the value reported by Gambacorta and colleagues using a LC-MS/MS method for simultaneous determination of ATs in spices and herbs from Lebanon (Gambacorta et al., 2019).

The positive incidence in edible and medicinal plant in the present study was consistent with previous reporting of ATs in these matrices (Gambacorta et al., 2019, EFSA Panel on Contaminants in the Food Chain, 2011). Moreover, the levels of ATs were different. The maximum level of TEA (2102.31 μg/kg) was much lower than the maximum level of TEA in spices (106792 μg/kg) and herbs (4868 μg/kg) as reported by Gambacorta and colleagues (Gambacorta et al., 2019). However, it was approximately 13-fold higher than that (163 μg/kg) reported for the food group ‘Herbs, spices and condiments’ published in the EFSA’s opinion (EFSA Panel on Contaminants in the Food Chain, 2011). A similar situation was found for the maximum level of AOH and AME in our study compared to the results of Gambacorta and colleagues. More specifically, results indicated 636.4 μg/kg and 306.0 μg/kg in spices and 64.2 μg/kg and 161.3 μg/kg in herbs, respectively. The maximum level of AOH, AME, and TEN in this paper was much higher than that in the EFSA’s reports (5.0 μg/kg for AOH, 1.0 μg/kg for AME and 3.0 μg/kg for TEN). However, it is worth noting that the food group ‘Herbs, spices and condiments’ included mainly samples of tomato ketchup.

Further, it can be inferred there was a possible association between the contamination rate and the herb matrixes (Table 3). The occurrence of ATs in fruits (69.23% in positive samples) was obviously higher than that in roots (Alpinia officinarum rhizoma), seeds (Coix seed and Cassiae Semen), and whole plant (Menthae Haplocalycis Herba), indicating that fungi might grow more favorably on the fruits of the herbs, which are rich in water and sugar. Longan Arillus was found to be free of ATs, and this may have resulted from the active chemical compounds of Longan Arillus that possess antifungal effects. Further investigations covering a large number of different types of herbs will be necessary to confirm the susceptible type (e.g., fruits).

## Conclusions

We have established for the first time an UPLC-ESI^+/-^-MS/MS method coupled to a modified QuEChERS extraction for the simultaneous determination of 7 *Alternaria* toxins—alternariol, alternariol mono methylether, altenuene, altenusin, tenuazonic acid, altertoxin Ⅰ, tentoxin—in edible and medicinal herbs. After extensive optimization of this approach, satisfactory recovery, favorable sensitivity, low limits of detection and accept matrix effect were all obtained. Then, the developed analytical method was successfully applied to determine the presence of these toxins in edible and medicinal herbs commonly used in China. The results suggested *Alternaria* toxins were ubiquitously found in edible and medicinal herbs with high contamination levels; moreover, that AME, AOH, and TEA were the major contaminants. Fruits of herbs seemed more likely to be contaminated with *Alternaria* toxins relative to other parts (e.g., root, seed). Thus, except for aflatoxin, the presence of *Alternaria* toxins should be of concern in edible and medicinal herbs. This proposed method could be used for continuous monitoring of *Alternaria* toxins in herbs, thereby reducing the health risk to consumers.

## CRediT authorship contribution statement

**Xiangsheng Zhao:** Conceptualization, Methodology, Data curation, Writing – original draft, Funding acquisition. **Dan Liu:** Methodology, Validation. **Xinquan Yang:** Resources. **Lei Zhang:** Writing – review & editing. **Meihua Yang:** Writing – review & editing, Supervision, Funding acquisition.

## Declaration of Competing Interest

The authors declare that they have no known competing financial interests or personal relationships that could have appeared to influence the work reported in this paper.
