# Supplementary material for: Detection of seven Alternaria toxins in edible and medicinal herbs using ultra-high performance liquid chromatography-tandem mass spectrometry
Source: Food Chem X. 2021 Dec 11;13:100186. doi: 10.1016/j.fochx.2021.100186 (PMC9039941; doi:10.1016/j.fochx.2021.100186)
Supplement: Supplementary data 1 [file mmc1.docx]

**Detection of seven *Alternaria* toxins in edible and medicinal herbs using ultra-high performance liquid chromatography-tandem mass spectrometry**

Xiangsheng Zhao ^1*^, Dan Liu ^1^, Xinquan Yang ^1^, Lei Zhang ^2^, Meihua Yang^1,3*^

^1^ *Key Laboratory of Resources Conservation and Development of Southern Medicine of Hainan Province & Hainan Branch of the Institute of Medicinal Plant Development, Chinese Academy of Medical Sciences and Peking Union Medical College, Haikou 570311, China;*

*^2^ School of Traditional Chinese Medicine, Guangdong Pharmaceutical University, Guangzhou 510006, China;*

^3^ *Institute of Medicinal Plant Development, Chinese Academy of Medical Sciences & Peking Union Medical College, Beijing 100193, China*；

*Corresponding author

Xiangsheng Zhao, Hainan Branch of the Institute of Medicinal Plant Development, Chinese Academy of Medical Sciences and Peking Union Medical College, NO. 4, Yaogu 4 Road, Xiuying District, Haikou, Hainan Province, 570311, P.R. China. Tel: +86-898-31589003. Fax: +86-898-31589010; [xiangshengzhao@hotmail.com](mailto:xiangshengzhao@hotmail.com) .

Meihua Yang, Institute of Medicinal Plant Development, Chinese Academy of Medical Sciences & Peking Union Medical College, No. 151 Malianwa North Road, Haidian District, Beijing 100193 P. R. China; Tel.: +8610 57833277; Fax: +8610 57833020; [yangmeihua15@hotmail.com](mailto:yangmeihua15@hotmail.com)

**S1. Sample preparation methods (additional information)**

***One-step extraction***

The sample powder (1.0 g) was accurately weighed into a 50 mL centrifuge tube, after which 5.0 mL of H_2_O was added. The tube was vortexed for 1.0 min and allowed to settle for 20 min. Afterward, 5.0 mL of 1.0% FA in ACN was added and vortexed for 10 min. The tube was subsequently centrifuged at 10,000 rpm for 5.0 min. After, 2 mL of supernatant was evaporated under a stream of nitrogen gas at 40°C. The residue was re-dissolved in 1 mL of 50% ACN and centrifuged at 13,000 rpm for 15 min. Finally, 3.0 µL of the resulting solution was injected into UPLC-MS/MS for analysis.

***HLB cleaning method***

Sample powder (1.0 g) was accurately weighed into a 50 mL centrifuge tube and 5.0 mL of H_2_O was added. The tube was vortexed for 1.0 min and allowed to settle for 20 min. Afterward, 5.0 mL of 1.0% FA in ACN was added and vortexed for 10 min. Then, 2 mL of the supernatant was gently loaded onto an Oasis HLB cartridge, which was pretreated with 3 mL of methanol and 3 mL of water. The cartridges were washed with 3 mL of water and dried for 10 min before elution with 3 mL of MeOH. The eluate was evaporated under a stream of nitrogen gas at 40°C. The residue was re-dissolved in 1 mL of 50% ACN and centrifuged at 13,000 rpm for 15 min. Finally, 3.0 µL of the resulting solution was injected into UPLC-MS/MS for analysis.

***QuEChERS-dSPE***

Sample powder (1.0 g) was accurately weighed into a 50 mL centrifuge tube and 5.0 mL of H_2_O was added. The tube was vortexed for 1.0 min and allowed to settle for 20 min. Afterward, 5.0 mL of 1.0% FA in ACN was added and vortexed for 10 min. Then, 2.0 g of MgSO_4_ and 0.5 g NaCl were added, followed immediately by vortexing for 1 min and centrifugation at 10,000 for 5 min. After, 1.0 mL of supernatant was transferred into a tube containing 100 mg of sorbent (PSA, -NH_2_, MCX, GCB or C_18_) and 200 mg of MgSO_4_ for cleanup. The mixture was vortexed for 1.0 min and then centrifuged (13,000 rpm) for 15 min. Finally, 3.0 µL of the resulting solution was injected into UPLC-MS/MS for analysis.


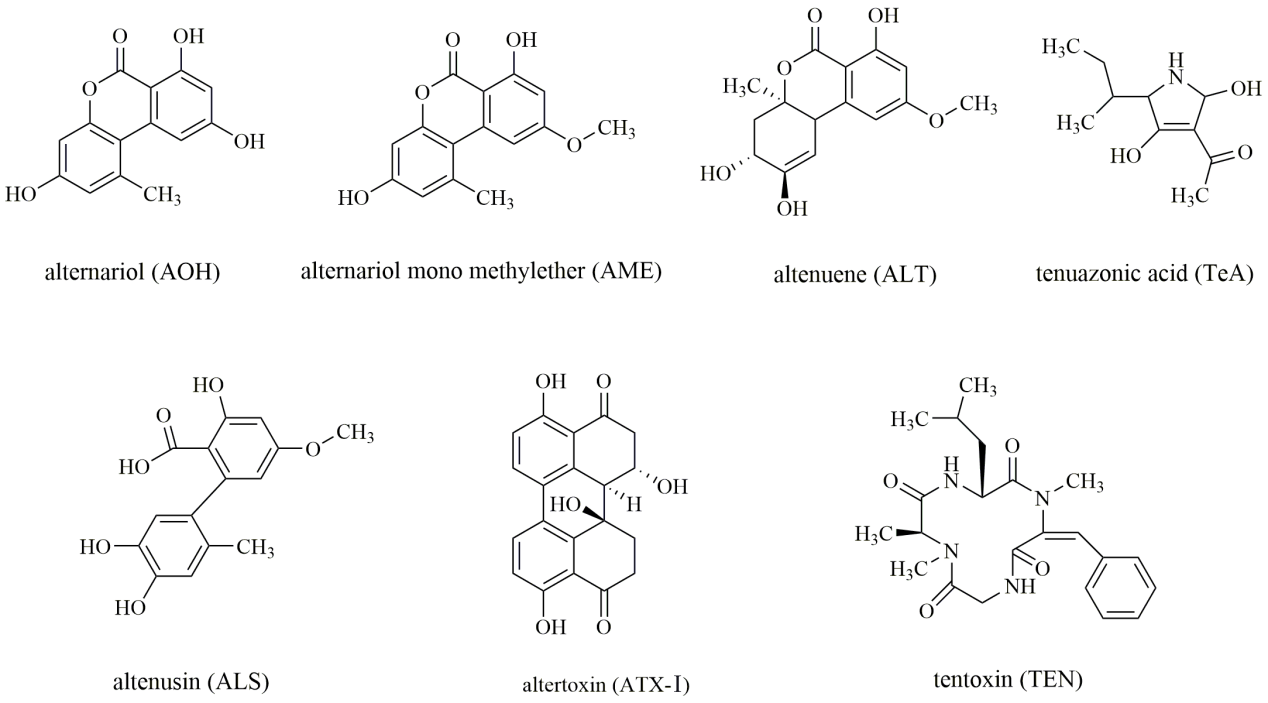


**Figure S1**. Chemical structures of the 7 *Alternaria* toxins.





**Figure S2**. Recoveries of SPE method for extraction of 7 toxins.





**Figure S3**. Effect of the amount of C_18_ on the recoveries of target mycotoxins.


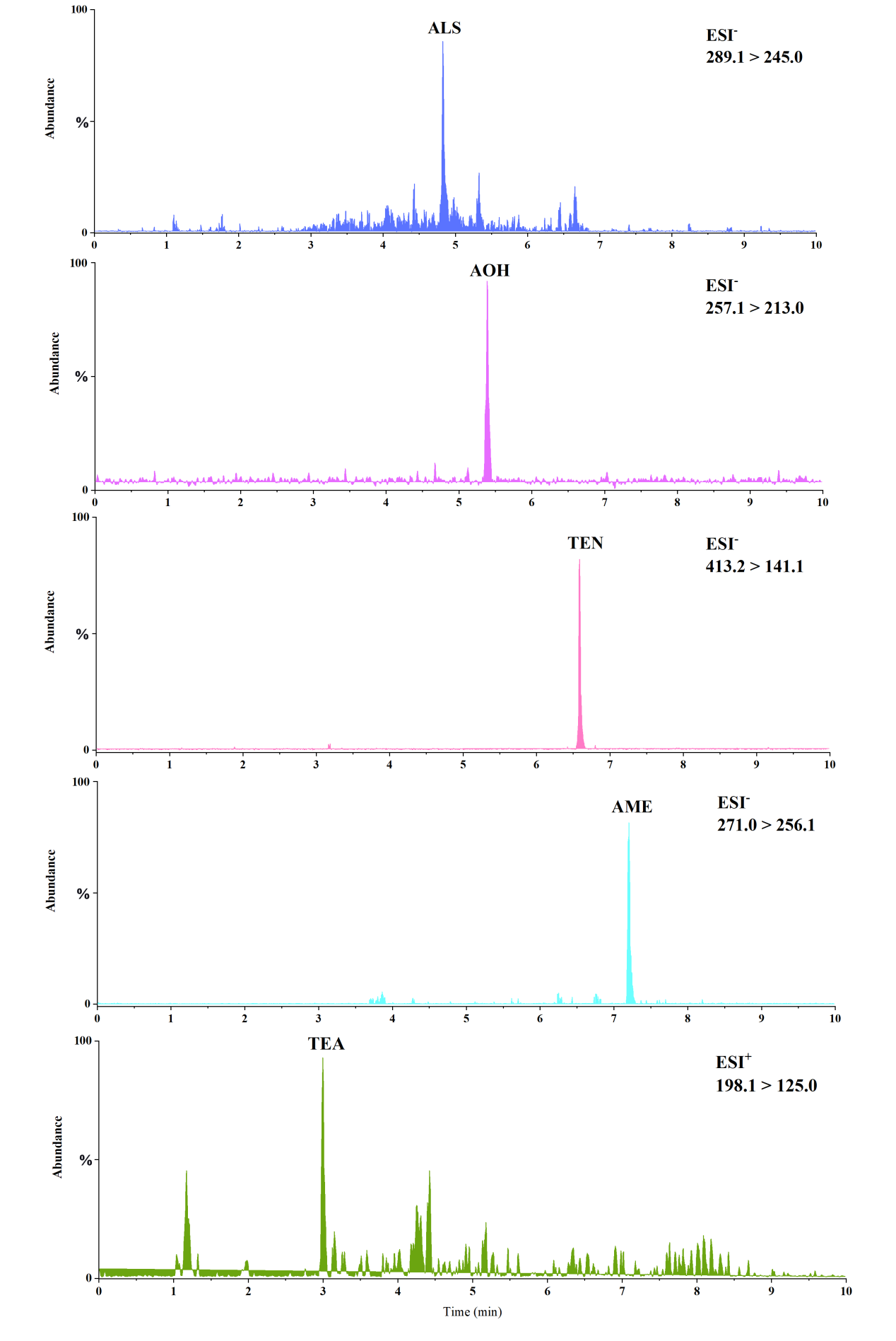


**Figure S4**. MRM chromatograms of positive Crataegi Fructus sample


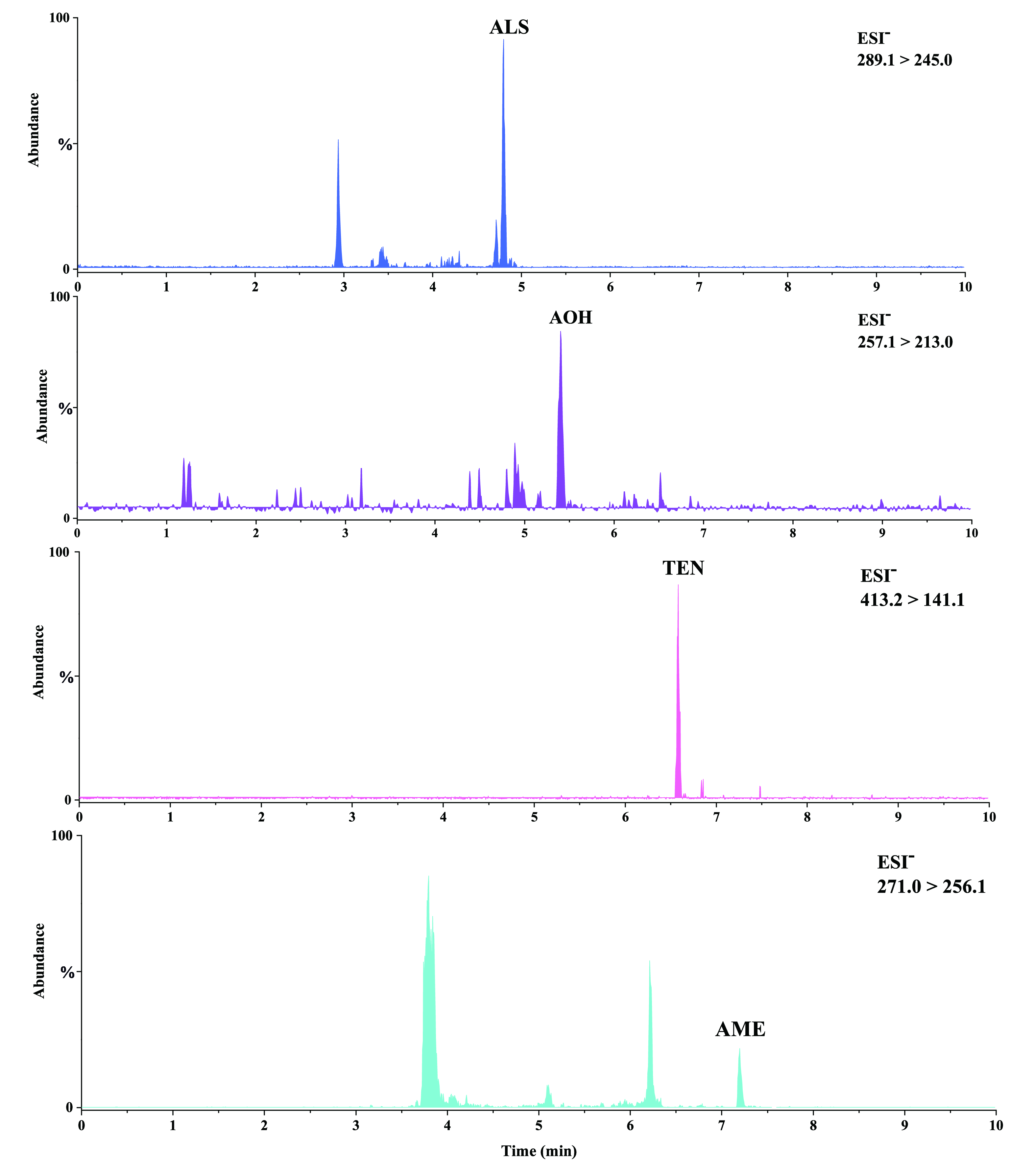


Figure S5. MRM chromatograms of positive Chaenomelis Fructus sample.


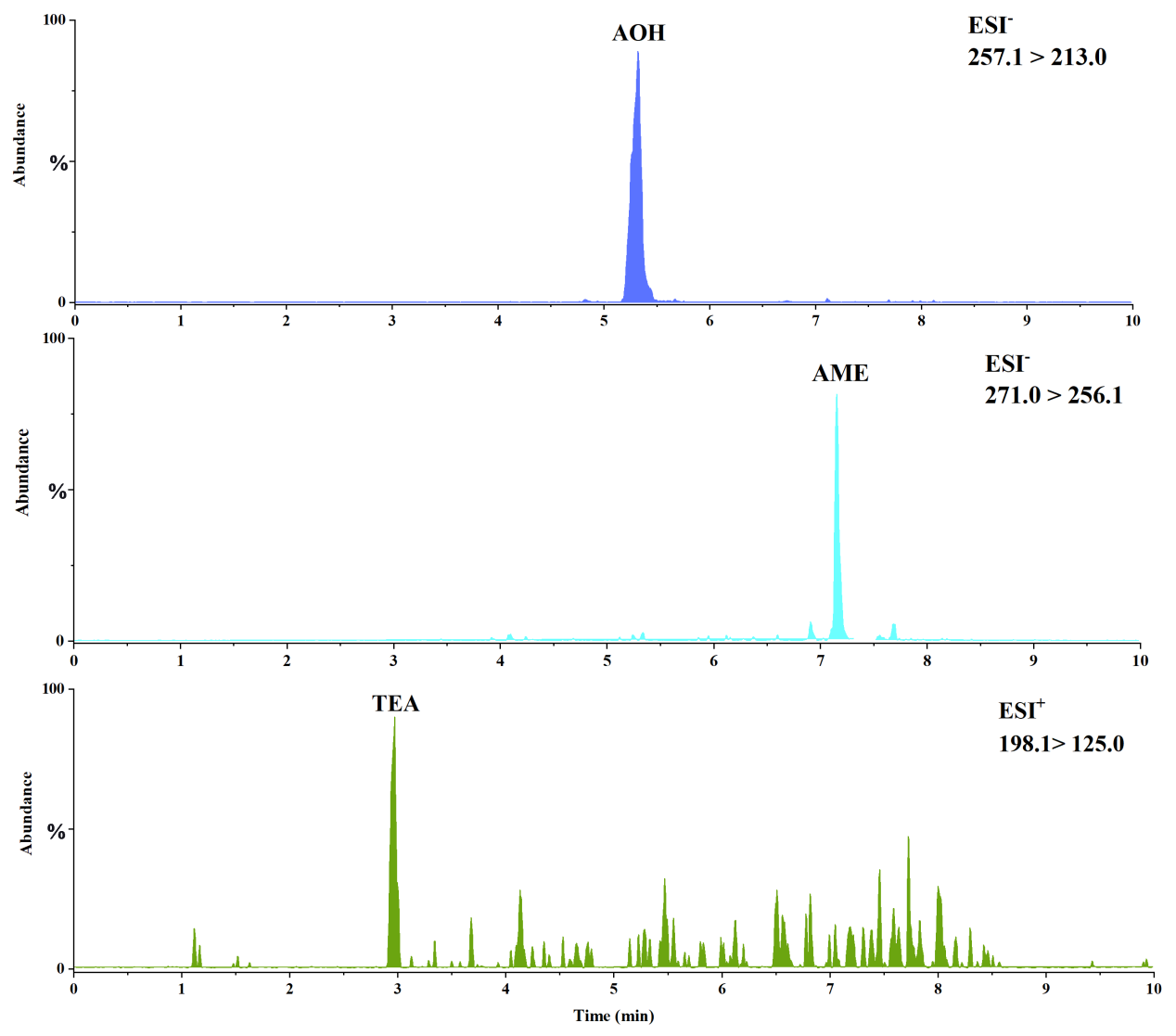


**Figure S6**. MRM chromatograms of positive Alpinia officinarum Rhizoma sample.


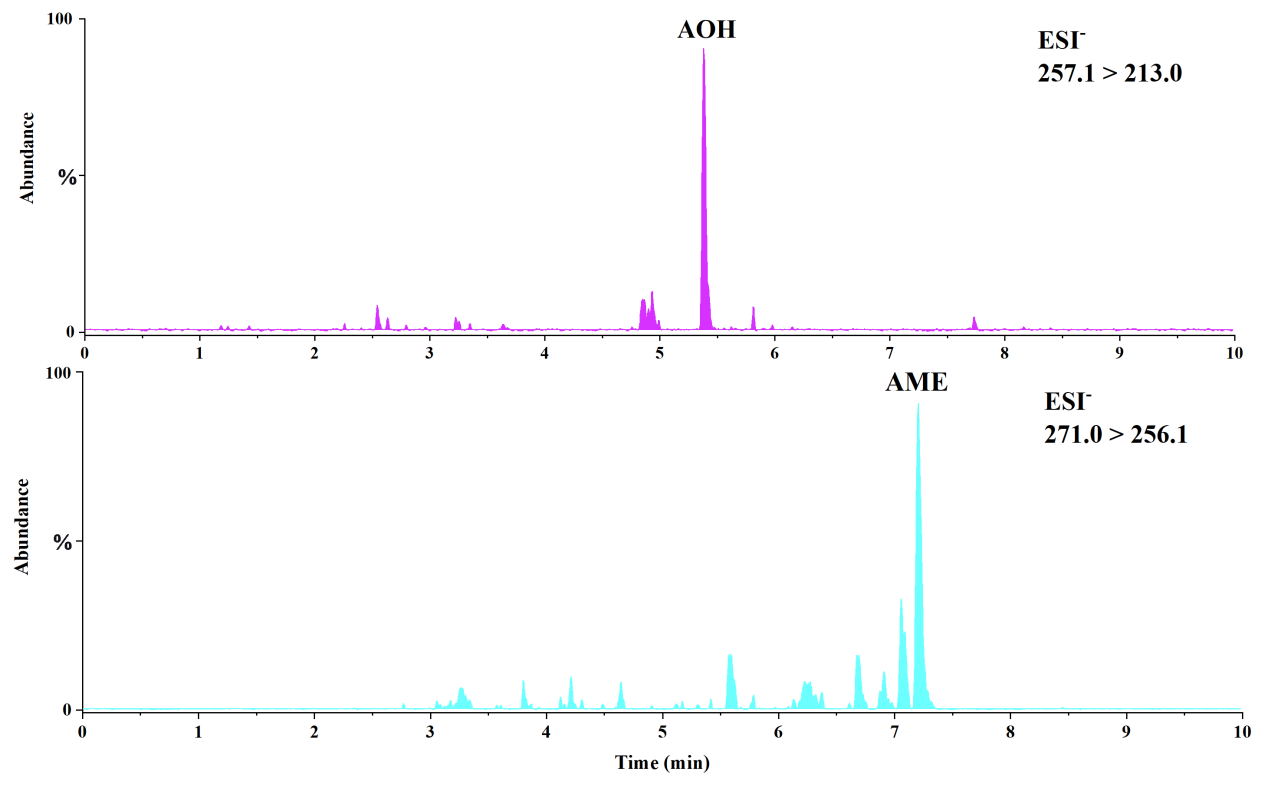


**Figure S7**. MRM chromatograms of positive Corni Fructus sample.


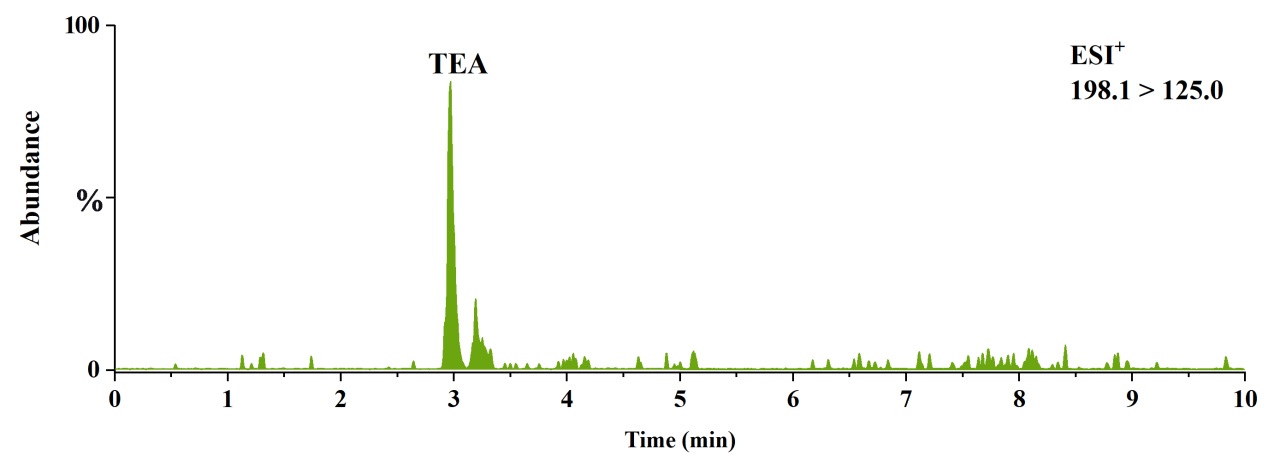


**Figure S8**. MRM chromatograms of positive Jujubae Fructus sample.


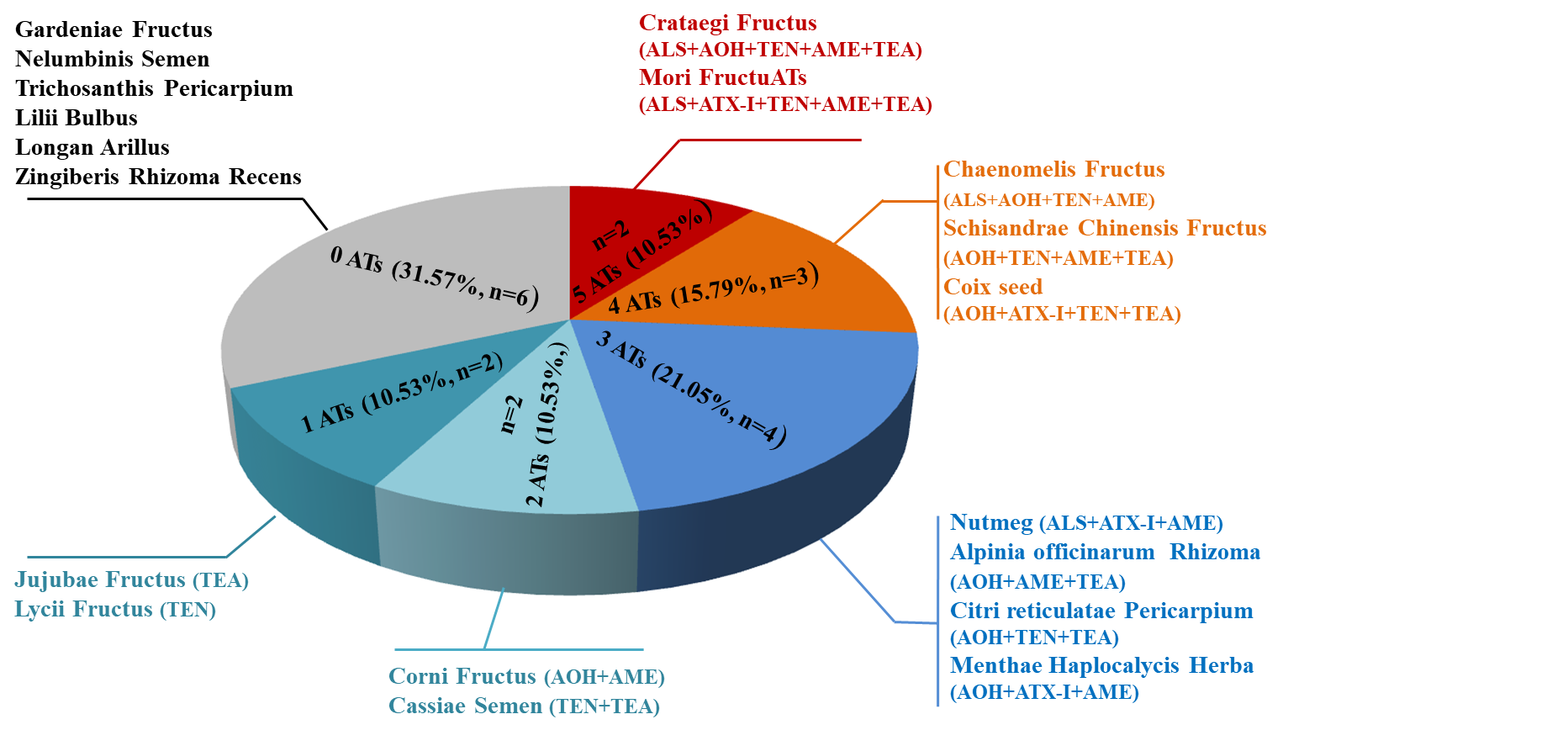


**Figure S9**. Alternaria toxins combinations in 13 edible and medicinal herbs.

**Table S1**. MS parameters for 7 *Alternaria* toxins and internal standards (IS)

| Toxins | RT  (min) | Ion  mode | Parent  ions(m/z) | Daughter  ion(m/z) | Cone  Voltage(V) | Collision  Energy(eV) |
| --- | --- | --- | --- | --- | --- | --- |
| ALS | 4.81 | ESI^-^ | 289.1 | 245.0 ^Q^ | 29 | 15 |
|  |  |  | 289.1 | 230.0 | 29 | 17 |
| AOH | 5.39 | ESI^-^ | 257.1 | 213.0 ^Q^ | 50 | 22 |
|  |  |  | 257.1 | 215.0 | 50 | 22 |
| ATX-Ⅰ | 6.28 | ESI^-^ | 351.1 | 315.0 ^Q^ | 37 | 17 |
|  |  |  | 351.1 | 333.0 | 37 | 10 |
| TEN | 6.59 | ESI^-^ | 413.2 | 141.1 ^Q^ | 40 | 17 |
|  |  |  | 413.2 | 271.1 | 40 | 15 |
| AME | 7.21 | ESI^-^ | 271.0 | 256.1 ^Q^ | 35 | 21 |
|  |  |  | 271.0 | 228.0 | 35 | 26 |
| TEA | 2.99 | ESI^+^ | 198.1 | 125.0 ^Q^ | 35 | 15 |
|  |  |  | 198.1 | 83.0 | 35 | 23 |
| ALT | 5.79 | ESI^+^ | 293.2 | 257.1 ^Q^ | 18 | 15 |
|  |  |  | 293.2 | 275.0 | 18 | 10 |
| AOH-IS | 5.37 | ESI^-^ | 271.3 | 226.1 ^Q^ | 50 | 24 |
|  |  |  | 271.3 | 156.0 | 50 | 34 |
| TeA-IS | 2.99 | ESI^+^ | 208.1 | 102.0 ^Q^ | 32 | 22 |
|  |  |  | 208.1 | 131.0 | 32 | 14 |

^Q^: transitions for quantification
